# Are active children and young people at increased risk of injuries resulting in hospital admission or accident and emergency department attendance? Analysis of linked cohort and electronic hospital records in Wales and Scotland

**DOI:** 10.1371/journal.pone.0213435

**Published:** 2019-04-10

**Authors:** Lucy J. Griffiths, Mario Cortina-Borja, Karen Tingay, Amrita Bandyopadhyay, Ashley Akbari, Bianca L. DeStavola, Helen Bedford, Ronan A. Lyons, Carol Dezateux

**Affiliations:** 1 Health Data Research UK, Wales and Northern Ireland, Swansea University Medical School, Swansea, United Kingdom; 2 Life Course Epidemiology and Biostatistics, UCL Great Ormond Street Institute of Child Health, London, United Kingdom; 3 Clinical Epidemiology, Nutrition and Biostatistics, UCL Great Ormond Street Institute of Child Health, London, United Kingdom; 4 Administrative Data Research Centre Wales, Swansea University Medical School, Swansea, United Kingdom; 5 National Centre for Population Health and Wellbeing Research, Swansea University Medical School, Swansea, United Kingdom; 6 Centre for Primary Care and Public Health, Barts and the London School of Medicine and Dentistry, Queen Mary University of London, London, United Kingdom; 7 Health Data Research UK London, Queen Mary University London, London, United Kingdom; Universite Cote d'Azur, FRANCE

## Abstract

**Introduction:**

Children and young people (CYP) are encouraged to increase time spent being physically active, especially in moderate and vigorous intensity pursuits. However, there is limited evidence on the prospective association of activity levels with injuries resulting in use of hospital services. We examined the relationship between objectively-measured physical activity (PA) and subsequent injuries resulting in hospital admissions or accident and emergency department (A&E) attendances, using linked electronic hospital records (EHR) from a nationally representative prospective cohort of CYP in Wales and Scotland.

**Methods:**

We analysed accelerometer-based estimates of moderate to vigorous (MVPA) and vigorous PA (VPA) from 1,585 (777 [46%] boys) seven-year-old Millennium Cohort Study members, living in Wales or Scotland, whose parents consented to linkage of cohort records to EHRs up until their 14th birthday. Negative binomial regression models adjusted by potential individual, household and area-level confounders, were fitted to estimate associations between average daily minutes of MVPA, and VPA (in 10-minute increments), and number of injury-related hospital admissions and/or A&E attendances from age nine to 14 years.

**Results:**

CYP spent a median of 59.5 and 18.1 minutes in MVPA and VPA/day respectively, with boys significantly more active than girls; 47.3% of children experienced at least one injury-related admission or A&E attendance during the study period. Rates of injury-related hospital admission and/or A&E attendance were positively associated with MVPA and VPA in boys but not in girls: respective adjusted incidence rate ratios (95% CI) for boys: 1.09 (1.01, 1.17) and 1.16 (1.00, 1.34), and for girls: 0.94 (0.86, 1.03) and 0.85 (0.69, 1.04).

**Conclusion:**

Boys but not girls who engage in more intense PA at age seven years are at higher risk of injury-related hospital admission or A&E attendance when aged nine to 14 years than their less active peers. This may reflect gender differences in the type and associated risks of activities undertaken. EHRs can make a useful contribution to injury surveillance and prevention if routinely augmented with information on context and setting of the injuries sustained. Injury prevention initiatives should not discourage engagement in PA and outdoor play given their over-riding health and social benefits.

## Introduction

The promotion of physical activity (PA) among children and young people (CYP) remains high on the agenda for policy makers and health professionals; however, despite public health efforts, many are insufficiently active with only half of 7-year-old children in the UK achieving the recommended 60 minutes of daily moderate to vigorous intensity PA (MVPA).[1] Girls are significantly less active than boys,[1] and activity levels decline during adolescence: nationally, only 19.7% of 13–15 year olds meet this daily MVPA guideline.[2]

The importance of being physically active is well documented, with significant and meaningful benefits, including reduced adiposity, favourable cardiometabolic profiles, improvements in musculoskeletal health, and psychological wellbeing.[3] Increased intensity of activity is associated with greater benefits; however, whilst the benefits of participation in PA are considered to outweigh the inherent risks,[4] injuries are one of the few potential adverse outcomes.

Injuries in CYP are a significant public health problem,[5] particularly in deprived communities,[6] and have individual, family and societal impacts.[7] Costs to the National Health Service (NHS) are also significant, estimated to be approximately £146 million a year, reflecting in part around two million visits each year to Accident and Emergency (A&E) departments alone for unintentional injuries in CYP.[8]

Current research reporting associations between CYP’s PA levels and risk of injuries is largely based on groups of CYP selected by their type of activity or injury.[9] Furthermore, studies have predominantly been based on parent-reported PA, injuries or both. To date, there have been fewer reports from studies examining objectively-measured PA levels in relation to subsequent injury-related hospital service utilisation in general population samples of CYP. We hypothesised that children engaging in higher intensity PA experience higher rates of injury-related hospital service utilisation in later childhood and early adolescence. We investigated this using electronic hospital records (EHRs) from Wales and Scotland linked to data from the Millennium Cohort Study (MCS), currently the largest contemporary child cohort study with nationally representative coverage.

## Methods

### Eligible study sample

The MCS is a prospective study of children born in the UK between September 2000 and January 2002. Infants who were alive and living in the UK at age 9 months were identified from child benefit registers. Disproportionately stratified sampling at electoral ward level ensured adequate representation of disadvantaged and ethnic minority areas.[10]

The first survey contact took place when the cohort child was around 9-months of age, when information was collected on 18,819 children (72% of those approached) through interviews in the home with the main (usually the mother) and partner respondents. Five further home interviews have been administered to date, at ages three, five, seven, 11 and 14 years. At each survey, information has been collected on demographic, social, and health factors related to the child and their family. At the age seven survey, adults with parental responsibility were also asked to give consent to link information collected within MCS to their child’s routine health records up until, but not beyond, their fourteenth birthday. Of the 13,681 singletons (children born singly, rather than one of a multiple birth) who participated in this survey, 1,951 and 1,598 were interviewed in Wales and Scotland, respectively; consent for record linkage was obtained for 3,304 children (1839 in Wales and 1465 in Scotland; 93% of those interviewed).

Linkage of EHRs to MCS cohort data was facilitated in Scotland by the NHS Information Standards Division (ISD), and in Wales using the Secure Anonymised Information Linkage (SAIL) Databank developed at Swansea University.[11–13] Linkage was achieved for 98.9% of the singletons whose parents provided consent, resulting in 3,269 children (1,838 in Wales and 1,431 in Scotland) with linked EHRs.

At the age seven survey, levels and patterns of PA were measured using the Actigraph GT1M uni-axial accelerometer (Actigraph, Pensacola, Florida), which measures acceleration in the vertical axis summed over a user defined time period (epoch) and is reported in the form of an activity count. This study used a 15-second sampling epoch. All children participating in the age seven interview were also invited to participate in the PA study. Children whose parents consented to their participation (1,838 in Wales and 1,500 in Scotland) were sent a programmed accelerometer and asked to wear it for seven continuous days during all waking hours, except when swimming or bathing. Data collection took place between May 2008 and August 2009. Reliable data (registered time of ≥10 hours on ≥2 days) were obtained from 1,659 children who were living in Wales (*n* = 898) or Scotland (*n* = 761).[14, 15] Information on predictors of consent for the accelerometry data and retrieval of reliable data in MCS is available elsewhere.[16]

**Fig 1:**
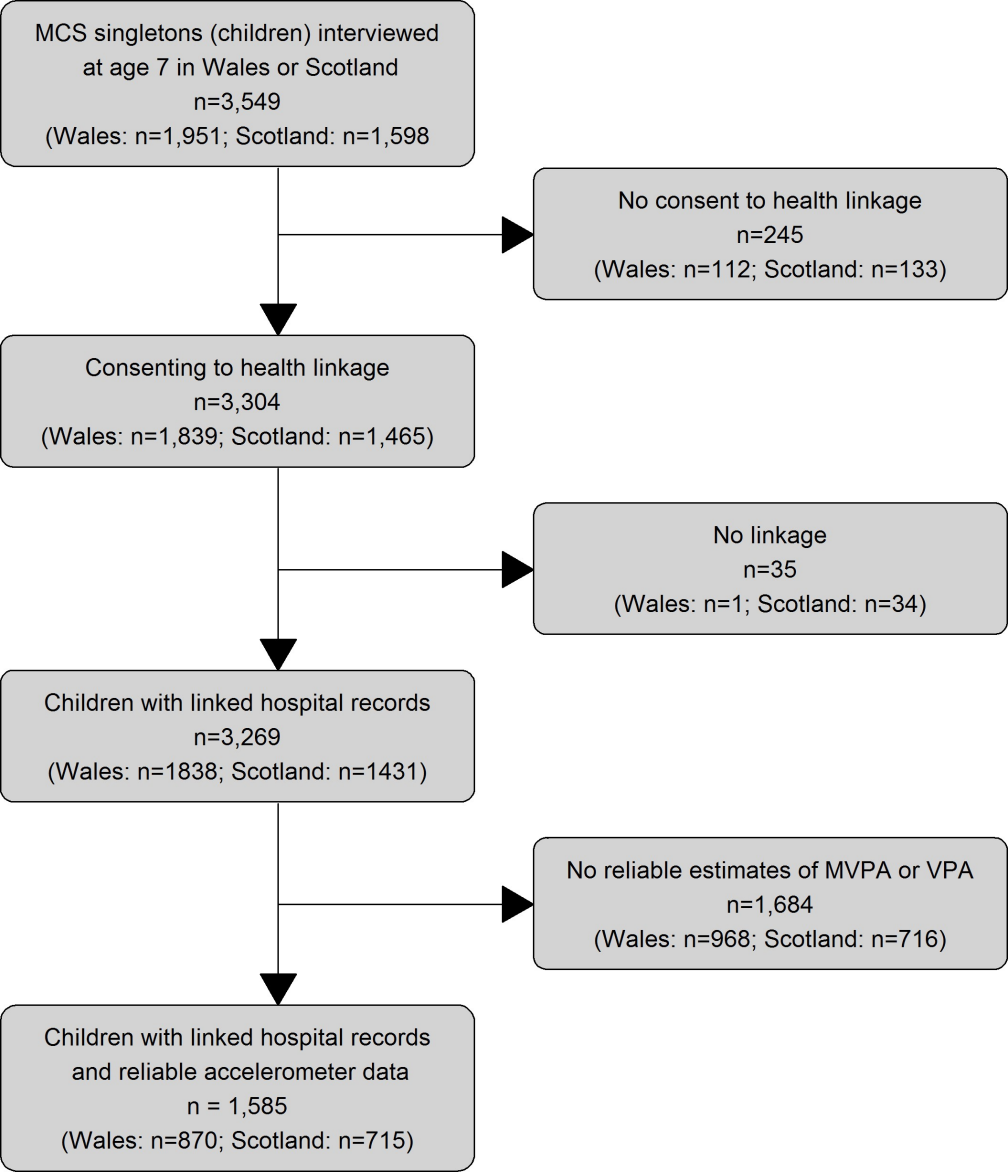
Flow diagram of study participants.

The study population eligible for these analyses comprised 1,585 children with estimates of daily activity based on reliable accelerometer data and linked hospital records (Fig 1).

### Primary outcome: Injury-related hospital admissions and A&E attendances

We analysed admissions and A&E attendances (AEAs) coded in EHRs for cohort members from age nine to 14 years and created a primary outcome combining injury-related AEAs.

We identified hospital admissions in a linked Patient Episode Database for Wales (PEDW) and Scottish Morbidity Records (SMR). We defined a hospital admission as a distinct Continuous Inpatient Spell (CIP), also known as a person superspell in SAIL; this is a definitive period of care within the NHS from admission to hospital to discharge (or death). It may contain one or more provider (organisation) spells, which may in turn contain one or more episodes (a period of care under one consultant). Readmissions within 48 hours were included in the same CIP. Both PEDW and SMR contain inpatient records and International Classification of Diseases 10th revision (ICD10)[17] diagnosis codes from 1997 and 1996 onwards, respectively.

We identified A&E attendances from the Emergency Department DataSet for Wales (EDDS), which contains a complete record of all emergency department activity in NHS Wales hospitals. Major (24-hour, consultant led) emergency departments started submitting information to the NHS Wales Informatics Service (NWIS) for merger into the EDDS from April 2009, whilst other hospitals providing emergency care facilities began submitting data from April 2012. For CYP living in Scotland, attendances were identified from the Scottish Accident and Emergency version 2 dataset (A&E2), which contains data from 2007 to the present day on patient attendances at Emergency Departments, Minor Injuries Units and community hospital A&Es across NHS Scotland.

We used data from 2009, the earliest year from when data was available from all four data sources. Since the MCS cohort was born in 2000–2001, outcomes were available from nine to 14 years of age. For each child, we counted the total number of injury-related AEAs over these five years of age. Injuries were identified using a comprehensive list of ICD10 diagnosis codes (S1 Table) and included if it was the primary diagnosis code in the admissions data or recorded anywhere on the A&E attendance record. Additionally, injury-related diagnoses were identified using EDDS and A&E local coding frameworks (S2 Table).[18, 19]

### Exposure variables: MVPA and VPA

Accelerometer data were used to derive, for each child, mean daily minutes of MVPA and vigorous physical activity (VPA), based on established accelerometer cut points (counts.min^−1^) of >2,240 to 3,840, and ≥3,841, for MVPA and VPA respectively. These cut-offs were chosen to optimise the discrimination between activity levels and were taken from our earlier calibration study in seven-year olds.[20]

### Covariates

Several potential confounding factors were identified *a priori* as associated with PA levels and/or AEAs for injuries in the literature and with attainable information in MCS. These included child factors: sex (obtained at the first MCS survey), long-standing illness (main survey respondents’ reports of any illness, disability or infirmity affecting the child over a period of time), weight status (based on measured weight and height, with derived body mass index [weight(kg)/height(m)^2^] categorised according to the UK1990 clinical reference standards [21] to group children into two categories: underweight/healthy weight and overweight/obese; maternal age at birth of cohort child; and household/area level factors: household income (dichotomised as at or above or below 60% of the national median using a modified Organisation for Economic Co-operation and Development (OECD) equivalence scale) and 2005 Rural/Urban Area Classification (RUAC). These were all measured at age seven years, except where indicated.

### Statistical analyses

Analyses were performed using STATA 15.0 (Stata Corporation, TX), and survey and non-response weights applied to account for the clustered sampling, attrition between contacts, consent to data linkage, and missing accelerometer data.

Negative binomial regression (NBR) models were used to account for overdispersion (relative to a Poisson distribution) in numbers of AEAs from age nine to 14 years and fitted to estimate incidence rate ratios (IRRs) for 10-minute increases in MPVA and VPA levels. These exposures were modelled separately, initially adjusting only for accelerometer wear time and sex, and then additionally for longstanding illness of child, weight status of child, household income, area of residence, maternal age at birth of cohort child. Interactions between each exposure (i.e. MPVA and VPA) and sex were examined, and sex-specific estimates reported when the latter were significant. All differences in proportions were tested using chi^2^ tests.

### Ethics approval and consent to participate

Ethical approval for the fourth survey of the Millennium Cohort Study was received from the Northern and Yorkshire Research Ethics Committee (07/MRE03/32). This study was approved by the Secure Anonymised Information Linkage (SAIL) Information Governance Review Panel (project 0410) in Wales and the Public Benefit and Privacy Panel for Health and Social Care (project 1617–0160) in Scotland. All data were anonymized prior to access and analysis.

## Results

### Sample characteristics

The majority of CYP in our sample (97.6%) were white, 45.6% were boys (Table 1). At age seven years, 21.3% had a longstanding illness and 16.6% were overweight or obese (Table 1). Just over a third were living in households with income poverty (defined as having a family income below 60% of the national median) and a third in rural areas. Overall, 22% of the respondents were lone parents/carers. The proportion of children with an injury-related AEA was similar for those with accelerometry data versus those without (47.3% vs 43.1% respectively; p = 0.34; Table 1).

**Table 1:** Characteristics of all children with linked hospital records and the study population for our analyses (children with reliable estimates of daily activity and linked hospital records).

	Children with linked hospital records *N* = 3269^1^ *n* (weighted %)	Children with reliable estimates of daily MVPA and VPA and linked hospital records *N* = 1585^2^ *n* (weighted %)
**Ethnicity of child**		
White	3,181 (97.4)	1,549 (97.6)
Non-white	83 (2.6)	34 (2.4)
**Gender of child**		
Male	1,678 (51.3)	777 (45.6)
Female	1,591 (48.7)	808 (54.4)
**Longstanding illness of child**		
No	2,647 (80.5)	1,318 (78.7)
Yes	618 (19.5)	266 (21.3)
**Weight status of child**		
Underweight/healthy weight	2,638 (82.8)	1,358 (83.5)
Overweight or obese	585 (17.2)	219 (16.6)
**Maternal age at birth of cohort member**		
14–19	305 (11.4)	82 (17.4)
20–29	1,472 (46.0)	668 (47.4)
30+	1,492 (42.6)	835 (35.1)
**Lone parent or carer**		
Non-lone	2,577 (77.8)	1,351 (78.1)
Lone	692 (22.2)	234 (21.9)
Household Income*		
Above median	2,361 (71.2)	1,244 (64.6)
Below median	907 (28.8)	341 (35.5)
**Living area**		
Rural	789 (25.7)	439 (30.9)
Urban	2,360 (74.3)	1,146 (69.1)
**Injury-related admissions/attendances 9–14 years**		
No	1,770 (56.9)	868 (52.7)
Yes	1,499 (43.1)	717 (47.3)

^1^Missing (*n* = 1,585): ethnicity (2); Longstanding illness of child (1); Weight status of child (8); academic qualification (3).

^2^Missing (*n* = 3269): ethnicity (5); Longstanding illness of child (4); Weight status of child (46); OECD (1); academic qualification (10).

All information collected at age 7 survey, except sex (collected at first survey).

*Defined using OECD 60% median poverty indicator

### Injury-related AEAs

Overall 47.3% of children experienced at least one injury-related AEA from 9–14 years, slightly more in Scotland than in Wales (47.8% (95% CI: 40.3%, 55.3%) (*n* = 304) and 45.5% (37.3%, 53.7%) (*n* = 413) respectively; *p* = 0.69). Boys were more likely to have at least one injury-related AEAs than girls (51.3% (95% CI: 44.4%, 58.1%) (*n* = 389) versus 43.9% (95% CI: 34.1%, 54.1) (*n* = 328) respectively), although not significantly more (*p* = 0.25).

The median (1^st^, 3^rd^ quartile) time spent in MVPA and VPA was 59.5 (46.4, 75.2) and 18.1 (12.5, 25.3) minutes/day, respectively. Boys were significantly more active than girls (*p*≤0.001 for MVPA and VPA); on average, they accumulated 66.8 (53.9, 82.9) and 20.8 (14.4, 28.8) minutes/day in MVPA and VPA, respectively, compared to girls who accumulated 53.3 (41.9, 67.1) and 15.96 (11.3, 22.2) minutes/day in pursuits of these intensities.

When we studied the association between MVPA and rates of injury-related AEAs, both the minimally adjusted and fully adjusted models indicated presence of effect modification by sex (*p* = 0.009 and *p* = 0.021, respectively; Table 2). The sex-specific estimates derived from these models are shown in Table 3 and indicate that, in boys, a 10-minute increment in MVPA is significantly associated with a 10% (according to the minimally adjusted model) or 9% (fully adjusted model) increase in rate of injury-related AEAs (fully adjusted IRR (95% CI): 1.09 (1.01, 1.17)). In girls, however, there was no support for an association in either the minimally or fully adjusted model (fully adjusted IRR (95% CI): 0.94 (0.86, 1.03)). A similar pattern was found for VPA, where the association between VPA and rates of injury-related AEAs was significant in boys (IRR (95% CI): 1.16 (1.00, 1.34)) but not in girls (IRR (95% CI): 0.85 (0.69, 1.04)). Although adjustment for the additional confounders did not seem to impact on the associations between PA and rates of injury-related AEAs, in the fully adjusted model there was a significant increase in rates in children with a reported longstanding illness than in those without (fully adjusted IRR (95%CI): 1.40 (1.09, 1.81); Table 2), and a significant decrease in rates in children living in rural as opposed to urban areas (IRR (95% CI): 0.69 (0.51, 0.92)). Since both longstanding illnesses and area of residence may have an impact on MVPA and VPA, although interesting, these estimates should be interpreted cautiously (and are highlighted here as an area for future investigation).

**Table 2:** Incidence Rate Ratios (IRR) and 95% confidence intervals (CI) derived from negative binomial regression models of association between moderate and vigorous physical activity (MVPA) and vigorous physical activity (VPA) at age 7 and subsequent injury-related hospital admissions and/or Accident and Emergency department attendances.

	Model 1^1^ Minimally adjusted		Model 2^2^ Fully adjusted	
	IRR (95% CI)	*p*-value	IRR (95% CI)	*p*-value
**MVPA (per 10 minute increment)** **	0.94 (0.85, 1.03)	0.171	0.94 (0.86, 1.03)	0.189
**Sex**				
Female	1 (base)		1 (base)	
Male	0.37 (0.18, 0.78)	0.010	0.42 (0.20, 0.90)	0.025
**Interaction of sex & MVPA**				
Male	1.17 (1.04, 1.32)	0.009	1.16 (1.02, 1.31)	0.021
**Longstanding illness of child**				
No			1 (base)	
Yes			1.40 (1.09, 1.81)	0.010
**Weight status of child**				
Underweight/Healthy weight			1 (base)	
Overweight/obese			1.09 (0.75, 1.58)	0.650
Household Income***				
Above median			1 (base)	
Below median			1.14 (0.81, 1.61)	0.449
**Area of residence**				
Urban			1 (base)	
Rural			0.69 (0.51, 0.92)	0.011
**Maternal age at birth of cohort member**				
14–19			1.16 (0.74, 1.81)	0.510
20–29			1.27 (0.99, 1.63)	0.060
30+			1 (base)	

Table Abbreviations: MVPA–moderate-to-vigorous physical activity; IRR–incidence rate ratio; CI–confidence interval

^1^Model 1 (minimally adjusted model): Adjusted for accelerometer wear time, sex, interaction term—sex × MVPA; *n* = 1,585.

^2^Model 2 (fully adjusted model): Adjusted for accelerometer wear time, sex, interaction term—sex × MVPA, longstanding illness of child, weight status of child, household income OECD 60% median poverty indicator, area of residence, maternal age at birth of cohort child; *n* = 1,576.

** Due to the significant MPVA × sex interaction term, the coefficients for MPVA and sex are to be interpreted as effects when, respectively, sex is female and MPVA is equal to 0.

*** Defined using OECD 60% median poverty indicator

**Table 3:** Incidence Rate Ratios (IRR) and 95% confidence interval (CI) derived from negative binomial regression models of association between moderate and vigorous physical activity (MVPA) and vigorous physical activity (VPA) at age 7 years and subsequent injury-related hospital admissions and/or Accident and Emergency department attendances for boys and girls separately.

	Model 1^1^ Minimally adjusted		Model 2^2^ Fully adjusted	
	IRR (95% CI)	*p*-value	IRR (95% CI)	*p*-value
**MVPA (per 10 minute increment)**				
Boys	1.10 (1.03, 1.17)	0.006	1.09 (1.01, 1.17)	0.019
Girls	0.94 (0.85, 1.03)	0.171	0.94 (0.86, 1.03)	0.189
**VPA (per 10 minute increment)**				
Boys	1.17 (1.02, 1.33)	0.024	1.16 (1.00, 1.34)	0.048
Girls	0.82 (0.66, 1.02)	0.071	0.85 (0.69, 1.04)	0.105

Table Abbreviations: MVPA–moderate-to-vigorous physical activity; VPA—vigorous physical activity; IRR–incidence rate ratio; CI–confidence interval

^1^Model 1 (minimally adjusted model): Adjusted for accelerometer wear time, sex, interaction term–sex × MVPA; *n* = 1,585.

^2^Model 2 (fully adjusted model): Adjusted for accelerometer wear time, sex, interaction term—sex × MVPA, longstanding illness of child, weights status of child, OECD 60% median poverty indicator, area of residence, maternal age at birth of cohort child; *n* = 1,576.

## Discussion

### Summary of main findings

We have found, in a nationally representative population of CYP living in Wales and Scotland, that around half were admitted to hospital or attended A&E departments for injuries, and that this was more common in boys than in girls. Boys who engaged in higher intensity physical activity, measured objectively at age seven years, had significantly increased rates of injury-related AEAs between nine and 14 years of age relative to boys who were less active at that age. This association was not found in girls.

### Strengths and limitations

To our knowledge, this is the first study in the UK to link objectively-measured PA obtained in a population sample of primary school children to their hospital records. Consent to linkage and linkage to hospital records were achieved in a very high proportion of children resulting in near complete population-based coverage of hospital records for the MCS cohort members living in Wales and Scotland.

The uniaxial accelerometers provided data on habitual activity levels over at least two days, but may have underestimated nonambulatory activities that did not involve vertical movement of the trunk (as they were waist mounted), such as cycling;[22] furthermore as they were removed during aquatic activities they did not include this type of PA. The data was processed to exclude unreliable data,[14, 15] and the thresholds used to define MVPA and VPA were derived from an earlier calibration study.[20]

Activity levels were however only measured over a single period at age seven years. We have assumed tracking of PA behaviour. Sporting activities tend to track through childhood,[23] although a recent analysis of pooled accelerometry-measured PA suggests gender differences in the stability of activity levels over time, with tracking of VPA most stable in boys with high baseline levels.[24] While we recognise that a follow-up measure of PA to assess activity levels over time would enable this assumption to be tested directly, repeat PA measures were not available in this study. Although the PA data was also only available for 48.5% of the children with linked EHRs, non-response weights for missing activity data were utilised, in addition to those used to address non-consent and attrition in the cohort.

We used NBR models in the data analyses to account for over-dispersion of count data. The breadth of information recorded in the MCS enabled adjustment for a wide range of potential confounding factors in analyses, although the potential for some residual confounding remains.

Our outcome comprised injury-related AEAs from age nine to 14 years, as these records were not available in both countries between age seven (time of PA measurement) and nine years. Our outcome did not include injuries managed in primary care, or by parents themselves, or in hospitals outside of Wales or Scotland. Whilst we described types of injuries coded, information on their severity or context was not available and the sample size precluded assessment with specific types of injuries.

### Existing evidence

The literature on injuries resulting from participation in sport is extensive, with the majority of studies examining selected populations, such as athletes, or CYP with selected clinical conditions.[25, 26] Nauta *et* al have reviewed the evidence on injuries associated with different physical activity behaviours, highlighting the risks of sports-related injuries among CYP arising from both organised and unorganised activities.[27] Most studies are cross-sectional in design and, unlike ours, include information based on self- or parent-reported behaviours and/or injuries.[9]

In line with our findings, existing evidence generally reports that boys have higher rates of unintentional injuries;[5, 28] however studies investigating sex differences in, specifically, physical activity -related injuries largely, although not consistently, report that girls are at greater risk.[27, 29] Childhood injuries resulting in healthcare utilisation associated with physical activity involvement in the general population have received little attention. Idler and colleagues[30] found no significant association between parental reported PA and healthcare utilisation in their cross-sectional study of 3,356 children aged 9 to 12 years. Similarly, Kirk and colleagues[31] found no association between self-reported PA and healthcare utilisation in 4,380 Canadian pupils aged 10 to 11 years. Although the latter studies used linked administrative, neither examined injury-related health service use specifically, or associations with intensity of physical activity.

### Implications for research, practice and policy

Our study has highlighted a small but significant increase in AEAs among boys related to increased intensity of activity. We were unable to examine context and nature of habitual activities, those resulting in injuries and types of injuries, and as a consequence our study provides no direct evidence on the basis of these findings to support limiting intensity of activity *per se*. On the contrary there is strong evidence for the benefits of higher intensity activity and the need to promote it in CYP. Instead we suggest specific guidelines are needed to prevent serious injury alongside efforts to engage more CYP in more PA.

Further research linking measures of PA and EHRs could therefore be enhanced with improvements in UK injury surveillance systems, which need to include more information on context and setting, better diagnostic data and more consistent monitoring.[19, 32–34] This will be aided, for example, by forthcoming datasets that provide more detailed information about how and why people attend A&E Departments, such as the Emergency Care Data Set.[35] Rapid developments in smartphone or wearable technology may also advance work to examine the favourable and potentially adverse outcomes of physical activity. Furthermore, use of these objective measures in combination with time-use diaries would provide greater understanding into the risk of injuries sustained during particular activities.

Gender differences in injury risk observed in our study may reflect gender differences in choice of active pursuits. There is some evidence that boys are more likely to take part in more physical, spontaneous or riskier play than girls. Boys also generally engage in more ‘risky’ recreational activities like football, cricket and rugby, which are associated with the highest non-fatal injury rates in the UK.[36] Qualitative work with CYP and their carers would provide helpful insights into the barriers to being physically active and, more specifically, into their perceptions of the risks and benefits inherent in different activities and how these influence amount and type of PA and risky outdoor play undertaken. This knowledge could inform advice and education about safety, ways to minimise serious unintentional injuries and, most importantly, the opportunity to educate on the benefits of being physically active and the importance of outdoor play, with its inherent risks, for healthy child development.[37]

Evidence from previous analyses of injury surveillance data from South Wales, suggests that 36% of fractures occurred during sport and recreation,[38] while an analysis of emergency department data from North Carolina found that 29% of unintentional injury-related visits among school-age youth were identified as sport and recreation-related injuries.[39] Injuries are however also clearly sustained from engagement in non-structured physical activities. Increased knowledge about different predictors of injuries, or risk of severe types, will inform the design and targeting of interventions aimed at preventing their occurrence amongst CYP and reducing associated inequalities. We need to understand how to maximise the health promoting and protecting elements of active childhoods while reducing risk of injury and recognise that they can never be completely eliminated.

It is important that prevention measures should promote and not discourage engagement in activities, and should include education of CYP to manage and reduce their risks of injury during activities.[40] Measures should focus on activities identified as having the highest risk of injury,[36, 41] with targeted interventions for CYP most likely to engage in them. For example, injuries sustained during rugby have received considerable attention, with recent calls for a ban on tackling during school rugby matches.[42, 43] Furthermore, it is important to prevent specific sport-related injuries, such as anterior cruciate ligament injuries, where evidenced-based methods, for example neuromuscular training programs,[44, 45] are available.

The National Institute for Health and Excellence (NICE) has published formal guidance on strategies to prevent unintentional injuries among CYP.[8] Recommendations include the modification of equipment and the environment, and the provision of information, education and safety equipment. However, like other agencies,[36, 46] NICE advocates for an approach of ‘risk-benefit assessment’ to enable government and local authorities to develop and extend opportunities for all CYP to be physically active. This is especially important for playground initiatives, which are also often at the forefront of injury prevention strategies despite the relatively low risk of injury in these environments.[46] Positively, the advocacy on play safety is therefore moving towards a more balanced approach, which will help CYP to play more freely in natural and urban environments.

## Conclusion

Moderate and vigorous PA is associated with increased risk of injury-related AEAs amongst boys, but not girls, living in Wales and Scotland, possibly reflecting gender differences in activities undertaken, associated risks and their settings, as well as the likelihood of being taken to hospital for an injury. Strategies are needed to prevent serious injury in CYP, while promoting their engagement in organised PA or play, and in the recognition and management of associated risks in order to ensure that the social and health benefits of being physically active outweigh the disbenefits occasioned through injury. Improvements in injury surveillance systems and further linkage studies using EHRs are needed to replicate our findings and to understand more about the context and severity of injuries sustained.

## Supporting information

S1 Table ICD10 injury codes. (DOCX)

S2 Table Emergency department local coding frameworks. (DOCX)
