# Supplementary material for: Are active children and young people at increased risk of injuries resulting in hospital admission or accident and emergency department attendance? Analysis of linked cohort and electronic hospital records in Wales and Scotland
Source: PLoS One. 2019 Apr 10;14(4):e0213435. doi: 10.1371/journal.pone.0213435 (PMC6457613; doi:10.1371/journal.pone.0213435)
Supplement: S1 Table — (DOCX) [file pone.0213435.s001.docx]

**S1 Table: ICD10 injury codes**

| Chapter XIX Injury, poisoning and certain other consequences of external causes | |
| --- | --- |
| Injuries to the head | S00-S09 |
| Injuries to the neck | S10-S19 |
| Injuries to the thorax | S20-S29 |
| Injuries to the abdomen, lower back, lumbar spine and pelvis | S30-S39 |
| Injuries to the shoulder and upper arm | S40-S49 |
| Injuries to the elbow and forearm | S50-S59 |
| Injuries to the wrist and hand | S60-S69 |
| Injuries to the hip and thigh | S70-S79 |
| Injuries to the knee and lower leg | S80-S89 |
| Injuries to the ankle and foot | S90-S99 |
| Injuries involving multiple body regions | T00-T07 |
| Injuries to unspecified part of trunk, limb or body region | T08-T14 |
| Effects of foreign body entering through natural orifice | T15-T19 |
| Burns and corrosions | T20-T32 |
| Frostbite | T33-T35 |
| Poisoning by drugs, medicaments and biological substances | T36-T50 |
| Toxic effects of substances chiefly nonmedicinal as to source | T51-T65 |
| Effects of air pressure and water pressure | T704, T708, T709 |
| Asphyxiation | T71 |
| Maltreatment syndromes | T741-T749 |
| Effects of other external causes | T751, T754, T79 |
| Chapter XX External causes of morbidity and mortality | |
| Pedestrian injured in transport accident | V01-V09 |
| Pedal cyclist injured in transport accident | [V10-V19](http://apps.who.int/classifications/icd10/browse/2016/en#/V10-V19) |
| Motorcycle rider injured in transport accident | [V20-V29](http://apps.who.int/classifications/icd10/browse/2016/en#/V20-V29) |
| Occupant of three-wheeled motor vehicle injured in transport accident | [V30-V39](http://apps.who.int/classifications/icd10/browse/2016/en#/V30-V39) |
| Car occupant injured in transport accident | [V40-V49](http://apps.who.int/classifications/icd10/browse/2016/en#/V40-V49) |
| Occupant of pick-up truck or van injured in transport accident | [V50-V59](http://apps.who.int/classifications/icd10/browse/2016/en#/V50-V59) |
| Occupant of heavy transport vehicle injured in transport accident | [V60-V69](http://apps.who.int/classifications/icd10/browse/2016/en#/V60-V69) |
| Bus occupant injured in transport accident | [V70-V79](http://apps.who.int/classifications/icd10/browse/2016/en#/V70-V79) |
| Other land transport accidents | [V80-V89](http://apps.who.int/classifications/icd10/browse/2016/en#/V80-V89) |
| Water transport accidents | [V90-V94](http://apps.who.int/classifications/icd10/browse/2016/en#/V90-V94) |
| Air and space transport accidents | [V95-V97](http://apps.who.int/classifications/icd10/browse/2016/en#/V95-V97) |
| Other and unspecified transport accidents | [V98-V99](http://apps.who.int/classifications/icd10/browse/2016/en#/V98-V99) |
| Falls | [W00-W19](http://apps.who.int/classifications/icd10/browse/2016/en#/W00-W19) |
| Exposure to inanimate mechanical forces | [W20-W49](http://apps.who.int/classifications/icd10/browse/2016/en#/W20-W49) |
| Exposure to animate mechanical forces | [W50-W64](http://apps.who.int/classifications/icd10/browse/2016/en#/W50-W64) |
| Accidental drowning and submersion | [W65-W74](http://apps.who.int/classifications/icd10/browse/2016/en#/W65-W74) |
| Other accidental threats to breathing | [W75-W84](http://apps.who.int/classifications/icd10/browse/2016/en#/W75-W84) |
| Exposure to electric current, radiation and extreme ambient air temperature and pressure | [W85-W99](http://apps.who.int/classifications/icd10/browse/2016/en#/W85-W99) |
| Exposure to smoke, fire and flames | [X00-X09](http://apps.who.int/classifications/icd10/browse/2016/en#/X00-X09) |
| Contact with heat and hot substances | [W00-W19](http://apps.who.int/classifications/icd10/browse/2016/en#/W00-W19) |
| Contact with venomous animals and plants | [W20-W49](http://apps.who.int/classifications/icd10/browse/2016/en#/W20-W49) |
| Exposure to forces of nature | [W50-W64](http://apps.who.int/classifications/icd10/browse/2016/en#/W50-W64) |
| Accidental poisoning by and exposure to noxious substances | [W65-W74](http://apps.who.int/classifications/icd10/browse/2016/en#/W65-W74) |
| Intentional self-harm | [X60-X84](http://apps.who.int/classifications/icd10/browse/2016/en#/X60-X84) |
| Assault | [X85-Y09](http://apps.who.int/classifications/icd10/browse/2016/en#/X85-Y09) |
| Event of undetermined intent | [Y10-Y34](http://apps.who.int/classifications/icd10/browse/2016/en#/Y10-Y34) |
| Legal intervention and operations of war | Y35-Y36 |
| Evidence of alcohol involvement determined by blood alcohol level / level of intoxication | Y90-Y91 |
| Chapter V Mental and behavioural disorders | |
| Mental and behavioural disorders due to psychoactive substance use – acute intoxication | F100, F110, F120, F130, F140, F150, F160, F170, F180, F190 |
