# Supplementary material for: Are active children and young people at increased risk of injuries resulting in hospital admission or accident and emergency department attendance? Analysis of linked cohort and electronic hospital records in Wales and Scotland
Source: PLoS One. 2019 Apr 10;14(4):e0213435. doi: 10.1371/journal.pone.0213435 (PMC6457613; doi:10.1371/journal.pone.0213435)
Supplement: S2 Table — (DOCX) [file pone.0213435.s002.docx]

**S2 Table:** Emergency Department local coding frameworks

Wales: Emergency Department DataSet for Wales (EDDS) local coding framework

| Variable name | Value | Meaning |
| --- | --- | --- |
|  |  | Wound |
| Diagnosis code | 03A | Laceration |
|  | 03B | Contusion |
|  | 03C | Abrasion |
|  | 03D | Soft tissue inflammation |
|  | 03Z | Wound, other or unspecified |
|  |  | Head Injury |
|  | 02A | Glasgow Coma Score 35 |
|  | 02B | Glasgow Coma Score <35 |
|  | 02C | Dental Injury |
|  | 02Z | Head Injury, other or unspecified |
|  |  | Fracture |
|  | 03A | Open Fracture |
|  | 03B | Closed Fracture |
|  | 03C | Fracture Dislocation |
|  | 03Z | Fracture, other or unspecified |
|  |  | Joint Injury |
|  | 04A | Sprain |
|  | 04B | Dislocation |
|  | 04C | Subluxation |
|  | 04Z | Joint Injury, other or unspecified |
|  |  | Amputation |
|  | 05Z | Amputation, other or unspecified |
|  |  | Soft Tissue Injury |
|  | 06A | Muscle Injury |
|  | 06B | Tendon Injury |
|  | 06C | Nerve Injury |
|  | 06D | Visceral Injury |
|  | 06E | Vascular Injury |
|  | 06Z | Soft Tissue Injury, other or unspecified |
|  |  | Burns, Scalds and Thermal Conditions |
|  | 07A | Electric |
|  | 07B | Chemical |
|  | 07C | Radiation |
|  | 07D | Scald |
|  | 07E | Sunburn |
|  | 07F | Hyperthermia |
|  | 07G | Hypothermia |
|  | 07H | Frostbite |
|  | 07Z | Burns, Scalds and Thermal Conditions, other or unspecified |
|  |  | Foreign Body |
|  | 08A | Ingested Foreign Body |
|  | 08Z | Foreign Body, other or unspecified |
|  |  | Puncture Wounds |
|  | 09A | Needle Stick Injury |
|  | 09B | Human Bite |
|  | 09C | Animal Bite |
|  | 09D | Insect Bite or Sting |
|  | 09Z | Puncture Wounds, other or unspecified |
|  |  | Poisoning or Overdose |
|  | 30A | Alcohol |
|  | 30B | Prescribed Drug |
|  | 30C | Non-prescribed/purchased drug |
|  | 30D | Illicit Drug |
|  | 30Z | Poisoning or Overdose, other or unspecified |
|  |  | Drowning |
|  | 33A | Near Drowning |
|  | 33Z | Drowning, other or unspecified |
| Diagnosis code |  | All ICD 10 codes listed in S1 Table |
| Treatment code |  | Wound Closure |
|  | 03Z | Wound Closure |
|  |  | Removal Foreign Body |
|  | 04Z | Removal Foreign Body |
|  |  | Manipulation |
|  | 06Z | Manipulation |

Scotland: Accident & Emergency (A&E) local coding framework

| Nature of injury | 01 | Wound |
| --- | --- | --- |
|  | 01A | Abrasion |
|  | 01B | Contusion includes bruise, haematoma |
|  | 01C | Blisters |
|  | 01D | Laceration |
|  | 01E | Incision |
|  | 01F | Needlestick |
|  | 01G | Bite animal |
|  | 01H | Bite human |
|  | 01J | Bite/sting insect |
|  | 01K | Flap laceration |
|  | 01L | Degloving wound |
|  | 01M | Penetrating wound |
|  | 01N | Skin avulsion |
|  | 01Z | Other |
|  | 02 | Bone and joint injury |
|  | 02A | Closed fracture |
|  | 02B | Open fracture |
|  | 02C | Dislocation of joint |
|  | 02D | Fracture dislocation |
|  | 03 | Soft tissue injury |
|  | 03A | Crush injury |
|  | 03B | Ligament avulsion |
|  | 03C | Ligament tear |
|  | 03D | Ligament rupture |
|  | 03E | Ligament strain |
|  | 03F | Muscle avulsion |
|  | 03G | Muscle tear |
|  | 03H | Muscle rupture |
|  | 03J | Muscle strain |
|  | 03K | Tendon dislocation |
|  | 03L | Tendon rupture |
|  | 03M | Tendon subluxation |
|  | 03N | Tendon strain |
|  | 03P | Tendon division |
|  | 03Q | Neuropraxia |
|  | 03R | Nerve compression |
|  | 03S | Complete transection of nerve |
|  | 03T | Partial division of nerve |
|  | 03U | Nerve contusion |
|  | 03V | Nerve entrapment |
|  | 03Z | Other Soft tissue injury |
|  | 04 | Head Injury |
|  | 04A | Concussion |
|  | 04B | Extradural Haemorrhage |
|  | 04C | Subdural Haemorrhage |
|  | 04D | Traumatic cerebral oedema |
|  | 04Z | Other head injury |
|  | 05 | Dental Injury |
|  | 05A | Avulsion |
|  | 05B | Fracture |
|  | 05Z | Other dental injury |
|  | 06 | Vascular Injury |
|  | 06A | Arterial haemorrhage |
|  | 06B | Arterial contusion |
|  | 06C | Arterial avulsion |
|  | 06D | Arterial perforation |
|  | 06E | Arterial rupture |
|  | 06F | Arterial transection |
|  | 06G | Venous haemorrhage |
|  | 06H | Venous contusion |
|  | 06J | Venous avulsion |
|  | 06K | Venous rupture |
|  | 06L | Venous transection |
|  | 06Z | Other vascula |
|  | 07 | Visceral injury |
|  | 07A | Traumatic haemothorax |
|  | 07B | Traumatic pneumothorax |
|  | 07C | Pulmonary contusion |
|  | 07D | Traumatic haemopneumothorax |
|  | 07E | Traumatic haemopericardium |
|  | 07F | Cardiac contusion |
|  | 07G | Pneumomediastinum |
|  | 07H | Rupture * |
|  | 07J | Perforation * |
|  | 07K | Avulsion * |
|  |  | * Refer ‘Bodily Location of Injury’ for specific visceral organs |
|  | 08 | Burn |
|  | 08A | 1st degree/duperficial |
|  | 08B | 2nd degree/partial thickness |
|  | 08C | 3rd degree/full thickness |
|  | 09 | Scald |
|  | 10 | Corrosion |
|  | 10A | 1st degree |
|  | 10B | 2nd degree |
|  | 10C | 3rd degree |
|  | 13 | Frostbite |
|  | 13A | Superficial |
|  | 13B | With tissue necrosis |
|  | 13Z | Other frostbite |
|  | 14 | Poisoning |
|  | 15 | Electric shock |
|  | 16 | Multiple injuries |
|  | 98 | Other nature of injury |
| Diseases code |  | All ICD 10 codes listed in S1 Table |
| Diagnosis code | 01 | Trauma/injury/poisoning |
| Procedure code | 01 | Wound care |
|  | 02 | Burn care |
|  | 03 | Limb immobilisation |
|  | 04A | Reduction of dislocation |
|  | 04B | Manipulation of fracture |
|  | 11A | Removal of foreign body from orifice |
